# Monte Carlo-based in-depth morphological analysis of medullary cavity for designing personalized femoral stem

**DOI:** 10.3389/fsurg.2024.1294749

**Published:** 2024-08-09

**Authors:** Lin Wang, Hui Sun, Kaijin Guo, Kunjin He, Weizhong Geng, Wen Zhou, Jian Wei

**Affiliations:** ^1^ School of Medical Information and Engineering, Xuzhou Medical University, Xuzhou, China; ^2^ Department of Orthopedics, Affiliated Hospital of Xuzhou Medical University, Xuzhou, China; ^3^ College of Internet of Things Engineering, Hohai University, Changzhou, China; ^4^ College of Computer and Information Engineering, XinXiang University, XinXiang, China

**Keywords:** femoral medullary cavity, region of interest, Monte Carlo method, morphological analysis, femoral stem

## Abstract

**Background:**

The design of femoral stem prostheses requires a precise understanding of the femoral marrow cavity. Traditional measurements of morphological parameters in the upper femur, particularly the medullary cavity and cortical region, are primarily based on coronal and sagittal axes, which may not fully capture the true three-dimensional structure of the femur.

**Methods:**

Propose a Monte Carlo-based method for a more comprehensive analysis of the femoral marrow cavity, using CT scans of femurs from a selected group of patients. The study aimed to define and calculate anatomically semantic morphological parameters to enhance the understanding of the femoral marrow cavity's anatomical morphological changes, ultimately improving the design and clinical selection of femoral stem prostheses. To enhance the accuracy of femoral stem prosthesis design, this study aims to develop a Monte Carlo-based method for a more comprehensive analysis of the femoral marrow cavity. The proposed method transforms the non-random problem of determining cross-sectional size into a random issue, allowing for the calculation of the size of the medullary cavity and cortical region. Anatomically semantic morphological parameters are then defined, calculated, and analyzed.

**Results:**

The experimental results indicate that the newly defined parameters complement existing ones, providing a more rational scientific basis for understanding the anatomical morphological changes of the femoral marrow cavity.

**Conclusion:**

This research offers essential scientific theoretical support for improved morphologic research, design, and clinical selection of femoral stem prostheses. It holds significant importance and application value in clinical practice, contributing to a more accurate and comprehensive understanding of femoral anatomy for prosthetic design.

## 1 Introduction

Hip arthroplasty is a surgical procedure in which the hip joint is replaced with a prosthesis to restore the movement of the hip joint (1). A prosthetic hip joint is mainly modeled after the human hip structure. By inserting the femoral stem prosthesis into the femoral marrow cavity of the patient, pain can be relieved while restoring range of motion and function (2, 3). Despite the continuous advancements in material science, prosthesis design, and surgical techniques, which have significantly reduced the incidence of complications following total hip arthroplasty, it is still not possible to completely avoid the need for revision surgery within five years post-operation (4). Therefore, ensuring the stability of the prosthesis is particularly crucial. Studies indicate that a good match and appropriate size between the femoral stem and the medullary cavity are crucial to achieving adequate initial stability (5), and they also effectively reduce the risk of intraoperative fractures of the femur during implantation (6–8). Consequently, improving the match between the femoral shaft and the medullary cavity has become an urgent issue to be addressed in hip surgery (9). However, the current situation is that the existing specifications and types of femoral stems are limited, and they cannot fully accommodate the needs of all patients. In clinical practice, there are still cases where inadequate anatomical matching between the prosthesis and the recipient bone leads to surgical failure (10–12).

Studies have shown that morphological parameters of the femoral marrow cavity are crucial to the design of the femoral stem (13, 14). Precise measurement and analysis of these morphological characteristics can enhance the adaptability, stability, and durability of femoral stem prostheses (15, 16). Therefore, in order to design a femoral stem prosthesis with the appropriate shape and size, it is necessary to conduct precise analysis of the femoral marrow cavity. However, the morphological characteristics of the femoral marrow cavity vary with age, gender, and region, among other factors (17, 18). This complexity has driven the development of various methods for measuring the morphological features of the femoral marrow cavity. For example, Zou et al. (19). used spiral CT to scan the entire length of the femur, studying the diameter and geometric shape of the marrow cavity in the upper femoral segment, which provided a basis for designing artificial femoral stems for the morphology of the Chinese population. Xue et al. (20). studied the statistical law of the geometric shape of the cross-section of the upper femoral marrow cavity and automatically extracted the cross-sectional shape of the upper femoral marrow cavity using computer image processing techniques, proposing a parametric mathematical method of conic curve fitting. However, although computer image processing methods can automatically extract the cross-sectional morphology of the marrow cavity and reduce human error, they may require complex algorithms and precise parameter settings. Additionally, computer-based measurement methods may not be applicable to all regions of the marrow cavity, especially in cases of abnormal or irregular morphologies.

**Figure 1:**
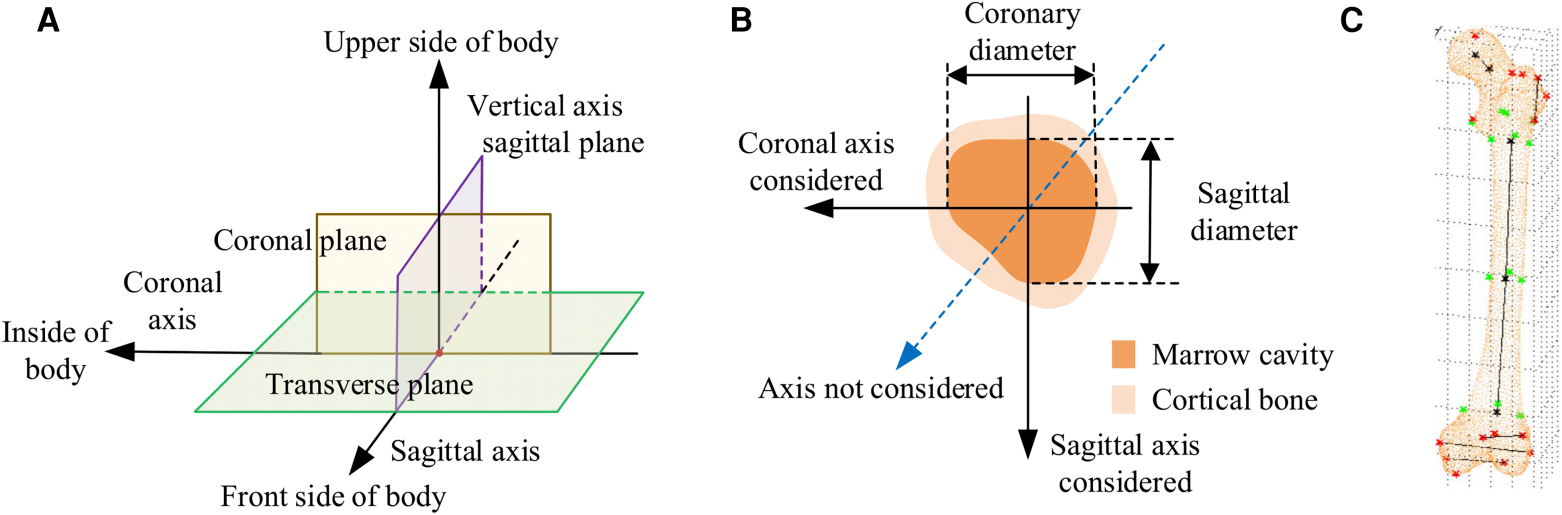
Current methods for measuring morphological parameters of the femoral marrow cavity. (**A**) 3D coordinate system of human anatomy. (**B**) Existing measurement method of cross section. (**C**) Geometric reference element.

In CT images, the normal anatomical morphology of the femoral marrow cavity shows a gradual decrease in diameter from proximal to distal, taking on a trumpet shape rather than a regular round shape. As illustrated in Figures 1A,B, current measurement procedures (21–26) have mainly focused on the coronal and sagittal axes, guided by anatomical markers (27, 28) as shown in Figure 1C. However, this approach ignores other axes, and measurement along the coronal or sagittal axes alone cannot fully or accurately reflect the true three-dimensional morphology of the femoral marrow cavity. Therefore, there is an urgent need for practitioners to gain a comprehensive understanding of the femoral marrow cavities in multiple dimensions.

In CT images, as shown in Figure 1B, the region of interest of the medullary cavity (referred to as ROIMC) includes the marrow cavity and the cortical bone area. The color contrast between the compact and spongy bone of the femur is strong, and the boundary betweenthem is clearly visible. This makes it easy to extract the marrow and cortical regions. The size of these irregular areas can be used as the basic unit to describe the cross-sectional morphology of the femoral marrow cavity. The Monte Carlo method has advantages in resolving the size of irregular areas. It is a computer numerical calculation method based on statistical sampling theory, which estimates and describes the statistics properties of functions through statistical sampling or random simulation of variables, and provides approximate solutions to problems (29). Due to its simple program structure, flexible simulation process, and adaptability in solving multidimensional problems, this method has been applied in many fields, including physics, engineering, and computer science. In clinical medicine, there have been reports of the application of the Monte Carlo method in calculation of radiation dosages (30). Kirby et al. (31) used the Monte Carlo method to generate a representative digital model that simulated the microstructural of trabecular bone. Eberlea et al. (32) established a non-destructive musculoskeletal simulation model based on kinematic data and used the Monte Carlo method to simulate non-contact anterior cruciate ligament injuries in the knee. Wang et al. (33) reported the use of an adaptive Monte Carlo method in top-down measurement assessment. By using the adaptive Monte Carlo method (aMCM), this study verified the effectiveness of the top-down method in the assessment of clinical laboratory uncertainties, which provides important theoretical support for the precise measurement of the femoral marrow cavity discussed in this study.

Based on years of research results obtained by our working group (34–36), this paper presents a Monte Carlo-based in-depth morphological analysis of the medullary cavity. Main work includes the extraction of the femoral ROIMC, Monte Carlo simulation of ROIMC, and calculation and analysis of femoral marrow cavity morphology. The research work can be used as a supplement to the existing research results, and therefore provide more reasonable scientific basis for comprehensively understanding the anatomical morphological changes of the femoral marrow cavity. Meanwhile, our research provides the necessary scientific theoretical support for improved morphologic research, design, and clinical selection of femoral stem prostheses and has important significance and application value in clinical practice.

The structure of the remainder of this study is as follows. Section 2 outlines the data sources and research methodology employed. Section 3 evaluates the reliability of the method proposed in this paper. Section 4 presents the experimental results. Section 5 discusses the findings. Finally, Section 6 concludes the study and suggests directions for future research.

## 2 Materials and methods

### 2.1 Data acquisition

The methodology presented in this work was verified using CT scans of femurs. In preparation, a total of 10 patients during 2019–2020 were voluntarily enrolled. Approval from the Ethics Committee of Xuzhou Medical University was obtained. Patients meeting one of the following requirements were excluded from our study: (1) individuals with a history of therapies that might potentially affect bone mass, structure; (2) women who were pregnant, breastfeeding, or who were planning to get pregnant; and (3) individuals with genetic relationships, such as parent-child or sibling relationships. After informed consent was obtained and associated risks were explained, their age, sex, height, medical history, and physical activity were obtained.

The CT images of these patients were scanned under the professional guidance of the clinician. Proper patient positioning is crucial for obtaining high-quality images in a CT scan. In this study, the patient was positioned as follows: The patient was supine in the center of the CT bed, with both lower limbs rotated in the neutral position and maintained in the standard anatomic position. To prevent the patient from moving during the scan, a mat was used to help keep the patient in position. Light Speed VCT helix scan, produced by GE, was used. The main parameters were as follows: tube voltage 120 kV; tube current 300 mA; layer thickness 0.6 mm; layer spacing 5.0 mm; scanning time 1.5 s. To facilitate parameter measurement, the imaging axis during the scan is set as follows: The imaging axis is defined relative to the patient's anatomy and the orientation of the CT beam. The main axis, shown in Figure 1A, is sagittal, dividing the body into left and right halves is sagittal from front to back, dividing the body into left and right halves. The coronal axis runs from side to side, dividing the body into anterior and posterior parts. The horizontal axis runs horizontally, dividing the body into upper and lower parts.

### 2.2 Methods

**Figure 2:**
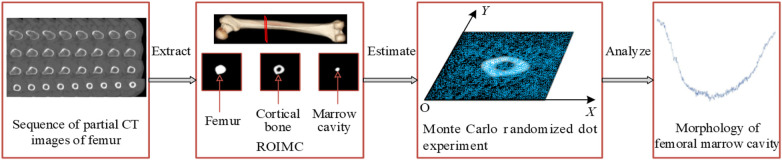
Research framework.

To obtain a more comprehensive understanding of femoral marrow cavity morphology and to improve the design quality of customized femoral stems, a morphological parameter analysis method based on Monte Carlo is proposed, as shown in Figure 2. The main steps of this framework are as follows:

Step 1: extract the ROIMC of the femur. The femoral marrow cavity and cortical bone region are extracted based on binarization, contour extraction, image subtraction, and other operations.
Step 2: calculate ROIMC statistics. According to the extracted medullary cavity and cortical bone, the statistics of medullary cavity and cortical bone are estimated based on Monte Carlo randomized dot experiment.
Step 3: define and calculate the morphological parameters of femoral marrow cavity. Taking the ROIMC as the basic unit and combining it with clinical needs, the morphological parameters of femoral marrow cavity are calculated.

#### 2.2.1 Extraction of ROIMC

The ROIMC mentioned in this paper mainly contains the cortical bone and medullary cavity areas on CT images. Considering that CT images are formed by the post-decay of electromagnetic rays through the human body, resulting in the presence of isolated pixel points or blocks with strong visual effects, unclear edge contours, and sharp changes in regional gray levels. Furthermore, the gray level of femur CT images is relatively complex, which increases the difficulty of contour extraction. Therefore, for denoising, Gaussian filtering is adopted to convolve the 2D Gaussian kernel with the CT femur image so that the value of each pixel can be obtained from the weighted average of its own and other pixel values in the neighborhood.

Gaussian filtering uses the product of two 1D Gaussian functions in the *x* and *y* dimensions, with their respective standard deviations, *σ_x_* and *σ_y_*, being equal. The formula is as follows:

(1) $$(\begin{array}{c} G(x,y)=\frac{1}{2\pi \sigma^{2}}e^{-(x^{2}+y^{2})/2\sigma^{2}}\;\\ u=u_{x}=u_{y}\;\\ \sigma =\sigma_{x}=\sigma_{y}\; \end{array}$$

On the basis of noise removal, the pixel value greater than the threshold is retained, while those with values less than the threshold, *T*_0_, are set to zero, as follows:

(2) $$dst(x,y)=\{\begin{array}{cc} src(x,y)\; & src(x,y)>T_{0}\;\\ 0\; & \mathrm{otherwise}\; \end{array}$$

**Figure 3:**
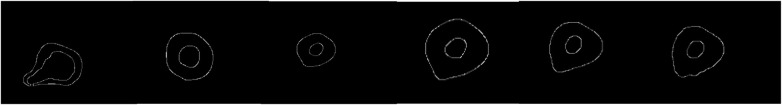
Detection of femoral medullary cavity and cortical bone boundaries based on the canny operator. Only a selection of key slices is shown here.

Additionally, while the high contrast between the cortical bone and the medullary cavity in CT imaging aids in distinguishing these structures, determining their precise boundaries remains challenging. To reduce potential biases, we employed the well-established Canny edge detection algorithm (37). This algorithm effectively eliminates some interference while precisely locating image edges. It achieves this by suppressing non-maximum values in the image and using a double-threshold approach for edge detection, which reduces the rate of edge misjudgment, sharpens the edges, and suppresses the appearance of false edges. In our experiments, we utilized the Canny operator, and the effect of boundary extraction on key slices is shown in Figure 3. By employing these methods, we have minimized biases that might arise in CT imaging studies, thereby enhancing the credibility and accuracy of our research findings.

**Figure 4:**
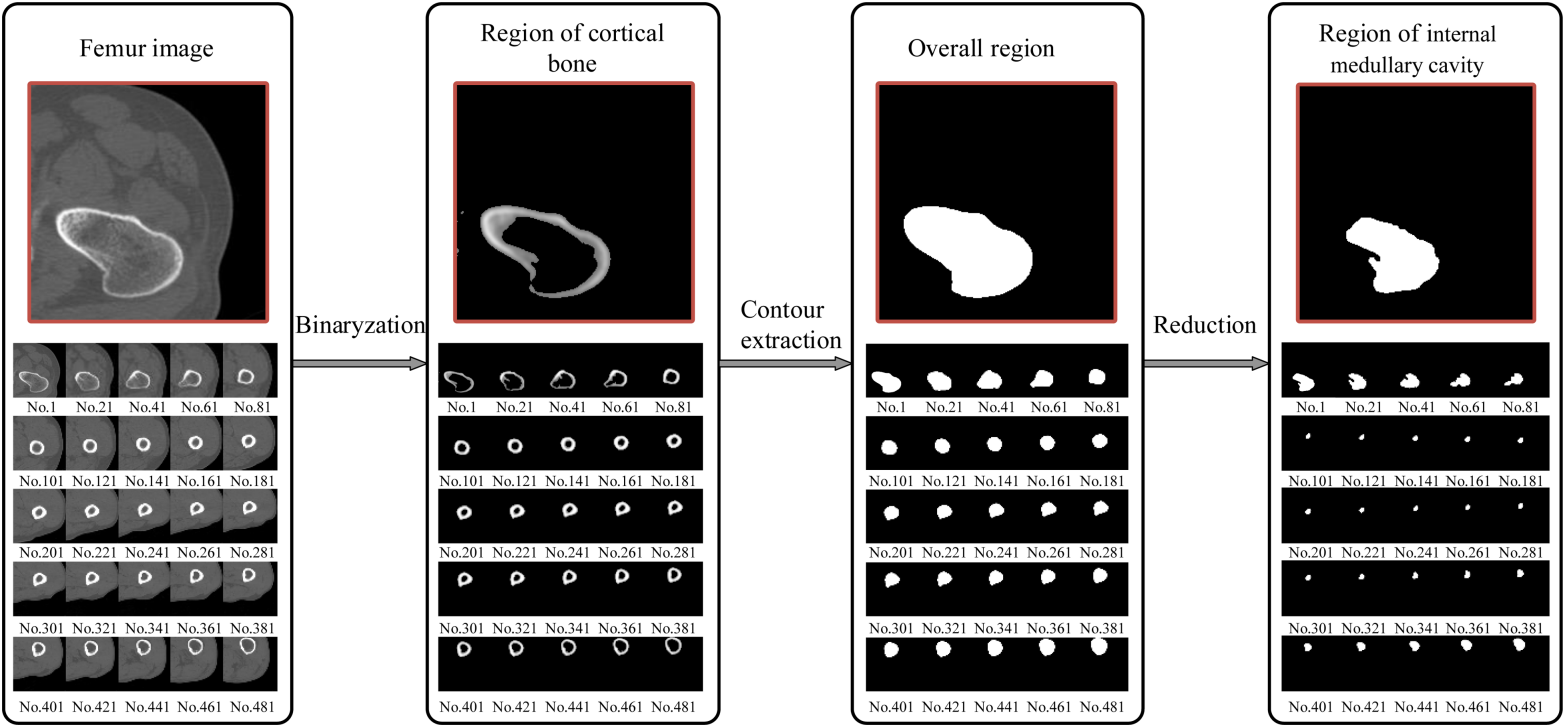
Extraction of the femur region of interest (ROI); from the proximal end to the distal end, one of every 20 CT images is selected. The final window size is 7 × 7, with a standard deviation (*σ*) of 3 and a threshold (*T*_0_) of 117. The size of the internal medullary cavity area, referred to as *s*_m_, describe the medullary cavity area in the transverse section. Similarly, the cortical area, eferred to as *s*_c_, describe the size of the cortical region in the transverse section.

Owing to the complexity of femoral CT images and the great influence of noise, by comparing the pixel values, contrast sizes, and actual characteristic information of different CT images in the femoral part, based on the precondition of ensuring the anti-noise effect and information integrity, *σ* and *T*_0_ are determined. The image is divided into different areas with gray characteristics by setting the appropriate gray threshold, and the cortical bone and medullary cavity are extracted through a series of operations, such as contour extraction and subtraction. The extraction result is shown in Figure 4.

#### 2.2.2 Calculation of ROIMC statistics

**Figure 5:**
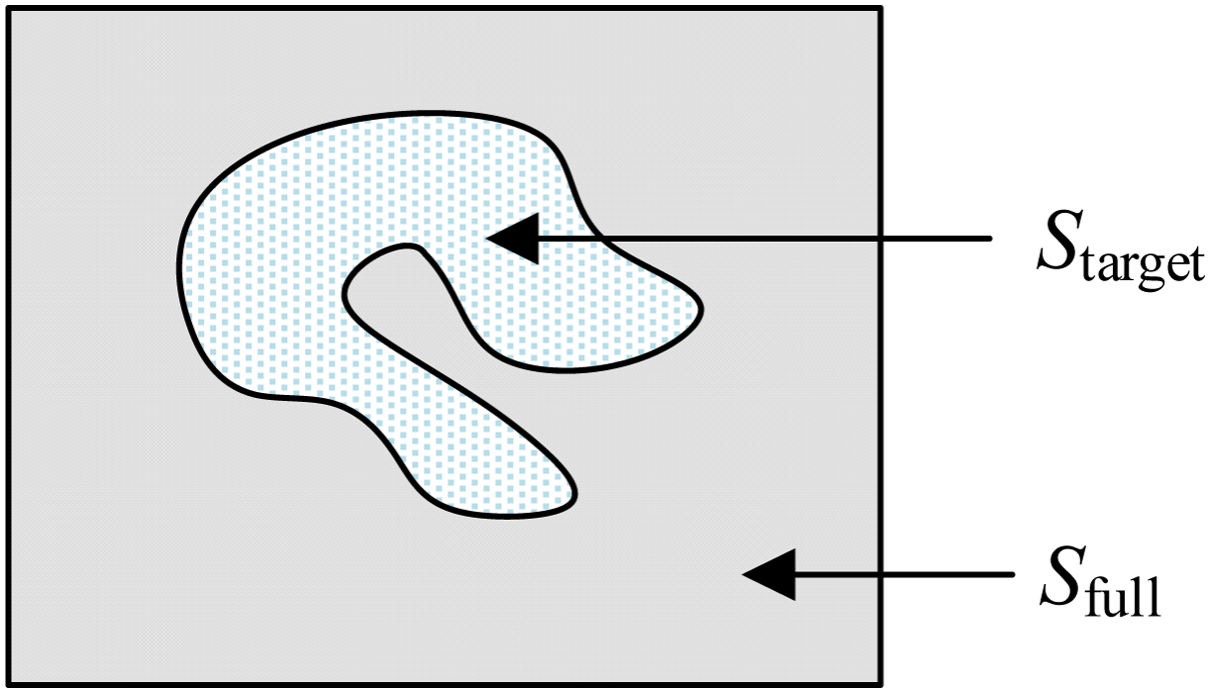
Irregular target area that can be computed using the Monte Carlo method.

Monte Carlo method establishe an appropriate probability model or a random process, set its parameters equal to the solution of the problem, and repeat sampling experiments until one obtain statistical analysis of the results. Monte Carlo method has unique advantages when solving a solution in an irregular region. Using the Monte Carlo method for calculating lake area is a very typical example. The area of the lake is determined from the number of balls fired randomly from a cannon that land in the lake. In relative terms, higher the number of balls fired is, better the estimation of the lake area is. Thus, the area of the lake is directly proportional to the number of balls that land in the lake. As is shown in Figure 5, the size of the lake (*S*_target_) is expressed as

(3) $$s_{\mathrm{target}}=S_{\mathrm{full}}.\frac{M_{\mathrm{target}}}{M_{\mathrm{full}}}$$

where *S*_full_ represents the size of the entire area, *M*_full_ represents the total number of balls, and *M*_target_ represents the number of balls that land in the lake.

**Figure 6:**
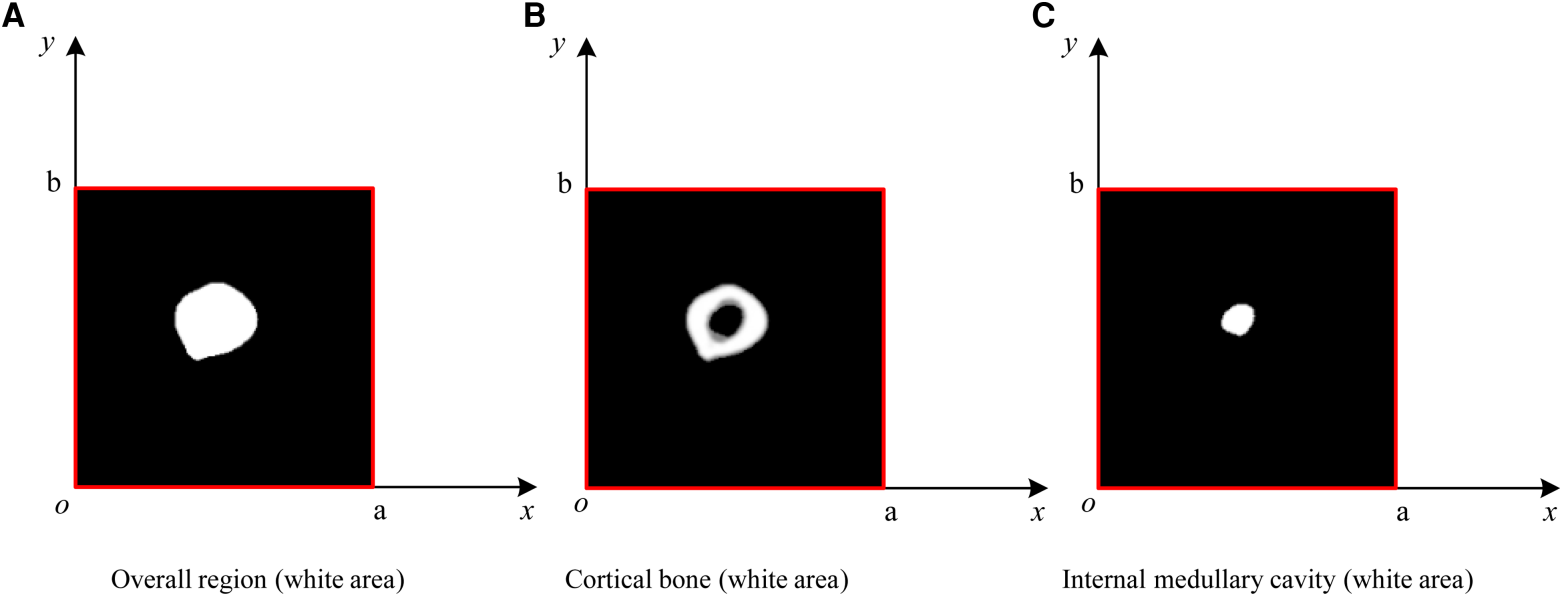
ROIMC simulation: the total number of random points is *n*; *t*he number of points falling into the femoral overall region in (**A**) is *n*_1_; the number of points falling into the black region in (**B**) is *n*_2_; the number of points falling into the cortical bone in (**b**) is *n* − *n*_2_; and the number of points falling into the internal medullary cavity in (**C**) is *n*_1_ − (*n* − *n*_2_).

In this paper, a probability model is established to describe the ROIMC in the form of statistics, and its estimated value is obtained via experimental simulation and mathematical statistics. As shown in Figure 6, *s*_m_ and *s*_c_ of a cross section of the diaphysis region, the main steps are as follows:

Step 1: set a rectangle of the same size as the femur CT image. The long side of the rectangle, *a*, is used as the *x*-axis, and the short side, *b*, is used as the *y*-axis to establish a Cartesian system. *a* and *b* are expressed in pixel. The intersection point of the long and short sides is the origin, *o*, of the coordinate system.
Step 2: conduct a simulation experiment. Random points having a uniform distribution of *X* and *Y* on [0, a] and [0, b], respectively, are generated in the rectangular region. The number of points falling into the area contained in the lateral boundary curve of the cortical bone (femoral overall area, white area in Figure 6A) is *n*_1_, and the number of points not falling into the cortical bone area (white area) is *n*_2_. Thus, the number of points falling into the cortical bone area in Figure 6B and the white medullary cavity area in Figure 6C are *n* − *n*_2_ and *n*_1_ − (*n* − *n*_2_), respectively. Assuming that the proportion of cortical bone region to rectangular region, *α*_c_, and the proportion of medullary cavity region to rectangular region, *α*_m_, is calculated as follows:
  (4) $$\{\begin{array}{c} \alpha_{c}=\left(n-n_{2}\right)/n\\ \alpha_{m}=\left(n_{1}-(n-n_{2})\right)/n \end{array}$$
Step 3: the estimated value of the cortical bone region, *s*_c_, and the medullary cavity region, *s*_m_, in the transverse section of the femur are calculated as follows:
  (5) $$\left\{ \begin{array}{c} s_{c}=\alpha_{c}\times a\times b\;\\ s_{m}=\alpha_{m}\times a\times b\; \end{array}\right..$$

#### 2.2.3 Definition of morphological parameters

**Figure 7:**
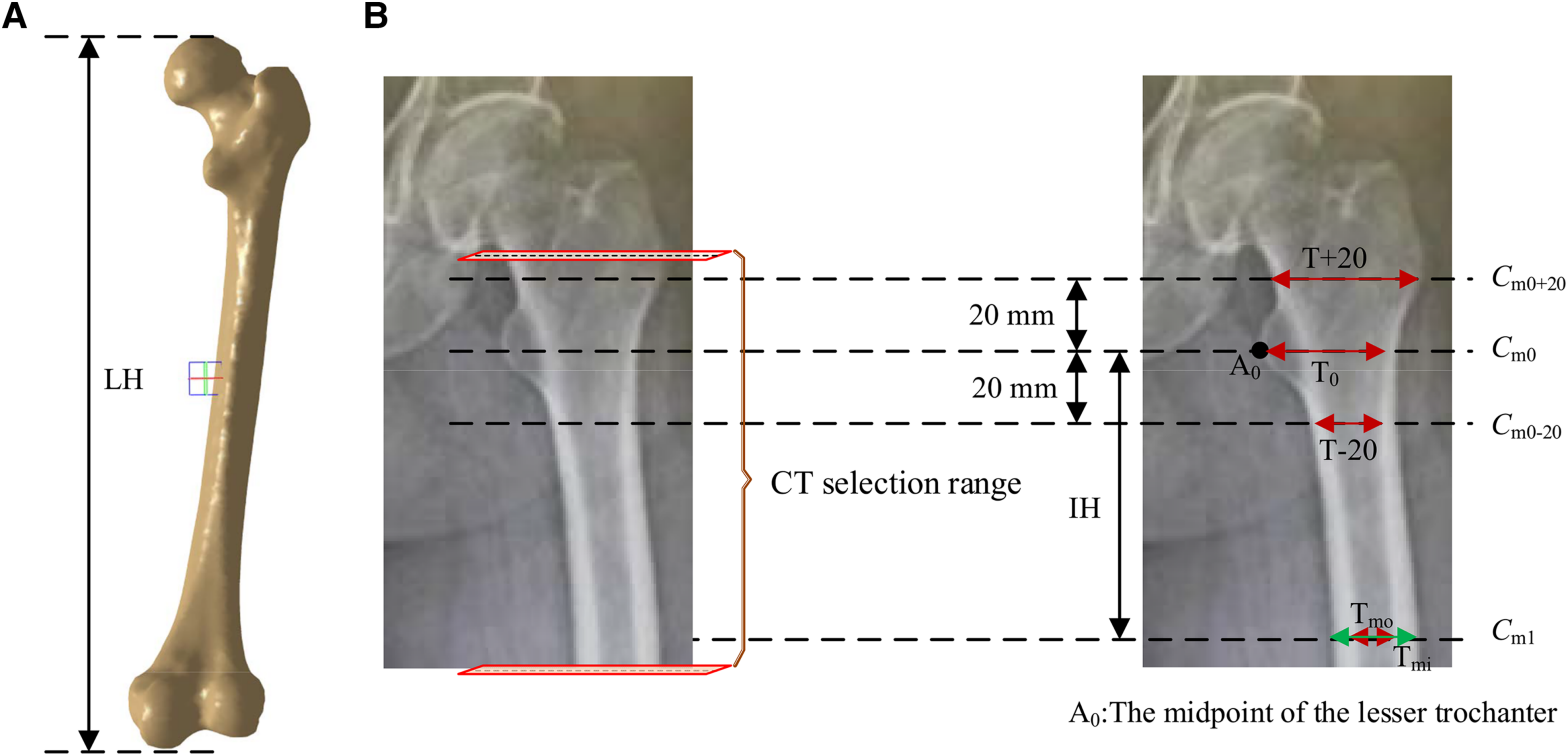
Key parameters defined based on the marked key cross sections. These mainly include the transverse section of midpoint of the lesser trochanter (the most prominent point of the proximal femur cross-sectional, *C*_m0_), the cross section 20 mm above the midpoint of the lesser trochanter (*C*_m0+20_), the cross section 20 mm below the midpoint of the lesser trochanter (*C*_m0-20_), and the cross section of the narrowest medullary cavity (cross-sectional femoral medullary cavity is the narrowest place, *C*_m1_). (**A**) Femur model. (**B**) The selection range of cross sections and marked key cross sections.

According to the requirements of the design of femoral prosthesis stem, it is necessary to combine clinical experience and data analysis to determine the range of CT data and the cross section of markers (38). As shown in Figure 7, in order to more accurately determine the cross section of the marker, it is necessary to expand the selection of more CT ranges. In addition, the labeling of key transverse sections is very important for the measurement of femur morphological parameters. The key sections of this paper refer to Noble's research (39).

According to the existing morphological parameters of the medullary cavity (18–22), the parameters listed in Table 1 are included in the calculation range. These parameters are roughly divided into three types: diameter parameters, length parameters and ratio parameters. The diameter parameters include the medullary internal diameter 20 mm above the lesser trochanter (*T* + 20), the medullary internal diameter 20 mm below the lesser trochanter (*T* + 20), the medullary internal diameter at the lesser trochanter (*T*_0_), the medullary internal diameter at the isthmus (*T*_mi_) and the medullary outside diameter at the isthmus (*T*_mo_). These parameters can be calculated based on key cross sections marked in Figure 7. The length parameters include isthmus height (IH) and the length of the femur (LH). The ratio parameters include the canal flare index (CFI), metaphyseal canal flare index (MCFI), distal canal flare index (DCFI), and cortical index (CI). These parameters are an important basis for the design of femoral stem prosthesis.

**Table 1:** Commonly used single-axial morphological parameters of personalized femoral marrow cavity.

Types	Parameters	Description (unit of the length parameter: mm; unit of the ratio parameter: 1)
Diameter parameters	*T* + 20	Medullary internal diameter 20 mm above the lesser trochanter
	*T* − 20	Medullary internal diameter 20 mm below the lesser trochanter
	*T*_0_	Medullary internal diameter at the lesser trochanter
	*T*_mi_	Medullary internal diameter at the isthmus
	*T*_mo_	Medullary outside diameter at the isthmus
	*T*_c_	The thickness of the cortical bone, with a thickness value on each slice.
Length parameters	IH	Isthmus height, the vertical height between the transverse plane where the center of the lesser trochanter is located and the transverse plane of the isthmus
	LH	The length of the femur, vertically, and the height between the highest and lowest point of the femur.
Ratio parameters	IH/LH	The ratio of IH to LH
	CFI	Canal flare index, and its value is equal to *T* + 20/*T_i_*
	MCFI	Metaphyseal canal flare index, and its value is equal to *T* + 20/*T* − 20
	DCFI	Distal canal flare index, and its value is equal to *T* − 20/*T_i_*
	CI	Cortical index, and its value is equal to (*T*_mo_ − *T*_oi_)/*T*_o_

In addition to the common morphological parameters listed in Table 1. According to the existing design requirements of femoral shaft prostheses, this paper defines the morphological parameters shown in Table 2. These parameters mainly include:

(a) *s*_c_, the size of cortical area, describe the size of cortical region in transverse section.
(b) *s*_m_, the size of the internal medullary cavity area, describe the of medullary cavity area in transverse section.
(c) *q*_1_, the ratio of the internal medullary cavity to the femoral overall region, describe the ratio of internal medullary cavity to femoral region on cross section.
(d) *q*_2_, the ratio of the internal medullary cavity to the cortical bone, describe the ratio of the internal medullary cavity to cortical bone region on cross section.
(e) *r*_1_, the growth rate of internal medullary cavity of the lesser trochanter, which describes the growth rate of internal medullary cavity region between *C*_m0−20_ and *C*_m0+20_. *s*_m0−20_ and *s*_m0+20_ represent sizes of internal medullary cavity area (pixel) at *C*_m0−20_ and *C*_m0+20_.
(f) *r*_2_, the growth rate of the internal medullary cavity of the upper femoral shaft, describes the growth rate of internal medullary cavity region between *C*_m1_ and *C*_m0+20_. *s*_m1_ and *s*_m0+20_ represent sizes of internal medullary cavity area (pixel) at *C*_m1_ and *C*_m0+20_.

**Table 2:** Morphological parameters of femoral marrow cavity.

Parameters	Description	Express formula
*s*_c_	Size of cortical area (pixel)	$s_{c}=\alpha_{c}.a.b$
*s*_m_	Size of internal medullary cavity area (pixel)	$s_{m}=\alpha_{m}.a.b$
*q*_1_	Ratio of internal medullary cavity to femoral overall region	$q_{1}=s_{m}/(s_{c}+s_{m})$
*q*_2_	Ratio of internal medullary cavity to cortical bone	$q_{2}=s_{m}/s_{c}$
*r*_1_	Internal medullary cavity growth rate of small trochanter	$r_{1}=\left(s_{m0+20}-s_{m0-20}\right)/s_{m0-20}$
*r*_2_	Internal medullary cavity growth rate of upper femur	$r_{2}=\left(s_{m0+20}-s_{m1}\right)/s_{m1}$

These parameters can be used to comprehensively examine all the dimensions of the femoral cross section and can reveal the change rule of the femoral marrow cavity from proximal to distal ends in a more comprehensive, detailed, and vivid way.

## 3 Rater reliability evaluation

Each patient's CT scan consists of over a thousand images. To verify the effectiveness and reliability of our method's computations on each slice, it is sufficient to select a single patient for validation. In this verification experiment, we chose a 50-year-old Han male patient with a height of 175 cm.

According to the design requirements of the femoral stem prosthesis, 492 DICOM format images (refer to the ranges marked in Figure 6B) were selected as experimental data. A Monte Carlo point experiment was implemented using Python v3.3 on a Windows-10 platform. The number of random points was 230,400, and its *X* and *Y* coordinates were integers between 0 and 239. Parameters shown in Table 4 were measured using Mimics v15.0 software. The processor was an Intel® Core™ i7-950H processor at 2.60 GHz, and the memory was 8 GB. SPSS v24.0 statistical software was used for statistical processing of the experimental data.

**Table 4:** ANOVA of *α*_mm_ and *α*_mt_, and *α*_cm_ and *α*_ct_.

Coefficient		Sum of squares	Degrees of freedom	Mean square	Significant
*α*_mm_ and *α*_mt_	Intergroup	0	1	0	0.999
	Intra-group	0	1	0	0.999
		0.320	982	0	/
*α*_cm_ and *α*_ct_	Intergroup	0	1	0	0.996
	Intra-group	0	1	0	0.996
		0.027	982	0	/

**Figure 8:**
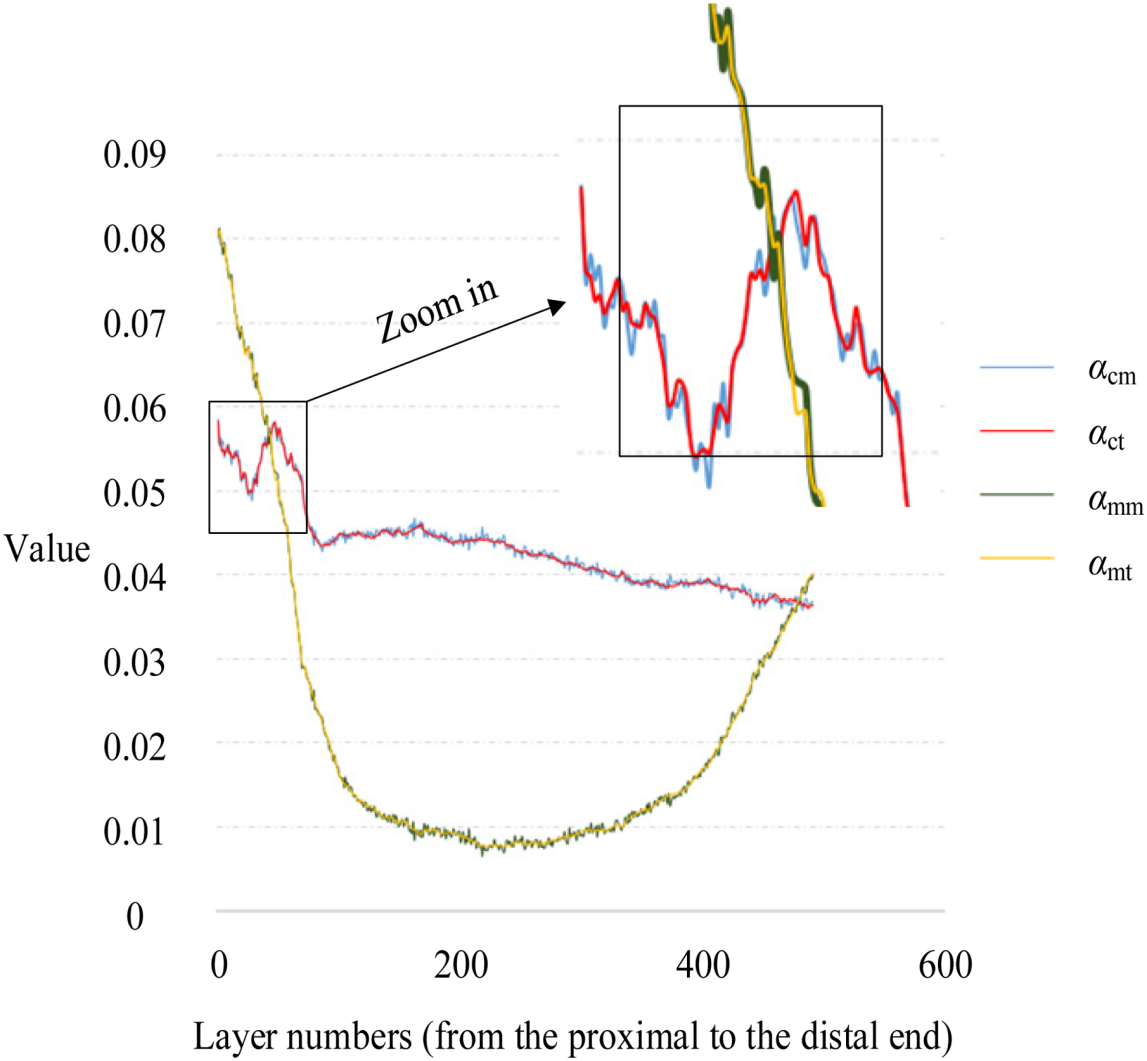
Variation of *α*_c_ and *α*_m_ with layer numbers.

To verify the accuracy of the Monte Carlo method, it was compared to the traversal method. In this experiment, traversal method refers to that each pixel in the target area of interest was accessed once and only once in turn along a search route. The target area of interest contains cortical bone and medullary cavity region. Two groups of parameters are compared. One group was *α*_cm_ and *α*_ct_, the proportion of cortical bone pixels in the whole CT image calculated by Monte Carlo method and traversal method, respectively. And the other group was *α*_mm_ and *α*_mt_, the proportion of medullary cavity pixels in the whole CT image calculated by Monte Carlo method and traversal method, respectively. The statistical descriptions of these parameters are shown in Table 3. Figure 8 shows the changes of these parameters on different layers. As can be seen from Table 3 and Figure 8, the results obtained by the Monte Carlo method are basically the same as those obtained by the traversal method.

**Table 3:** Statistical description of *α*_mm_, *α*_mt_, *α*_cm_, and *α*_ct_.

	Total layers	Average	Standard deviation	95% CI for the mean		Minimum value	Maximum
				Lower limit	Upper limit		
*α*_mm_	492	0.021415	0.0180375	0.019817	0.023013	0.0072	0.0809
*α*_mt_	492	0.021417	0.0180652	0.019816	0.023017	0.0076	0.0809
*α*_cm_	492	0.043364	0.0052359	0.042901	0.043828	0.0358	0.0586
*α*_ct_	492	0.043363	0.0052236	0.042900	0.043826	0.0360	0.0584

To further verify the correctness of this conclusion, a single-factor ANOVA test was adopted, and the results are shown in Table 4. As can be seen, the significance levels of *α*_m_ and *α*_c_ obtained by the Monte Carlo and traversal methods was 0.999 and 0.996, respectively, when the significance level of 0.05 was selected. This indicates that there was no significant difference between the two datasets. Therefore, using the Monte Carlo method to calculate ROIMC is reliable and feasible.

## 4 Results

### 4.1 Common morphological parameters

The built-in Measure Distance function in Mimics 15.0 (Materialize, Leuven, Belgium) (accuracy: 0.01 mm) was used to record the medullary internal diameter parameters shown in Table 1, and each measurement was repeated three times and then averaged. The diameter parameters as shown in Table 1 were measured as shown in Table 5. Meanwhile, the ratio parameters are also caculated according to the diameter parameters. Additionally, we have listed the corresponding parameter results from literature (19) and (21). It can be observed that the data in this paper are largely consistent with these research findings.

**Table 5:** Commonly used single-axial morphological parameters of personalized femoral marrow cavity.

Parameters	Values			
	Our work	Chen HX (23)	Pi YG (25)	Noble (39)
*T* + 20	43.24	39.9 ± 4.7	43.55 ± 5.26	45.4 ± 3.6
*T* − 20	18.07	17.2 ± 2.9	18.32 ± 2.98	21.0 ± 2.4
*T*_0_	24.40	/	25.47 ± 3.6	29.7 ± 2.9
*T*_mi_	10.57	11.3 ± 2.6	10.71 ± 2.26	12.1 ± 1.5
*T*_mo_	25.20	/	27.42 ± 2.8	/
IH	110.40	105.0 ± 15.9	106.97 ± 15.92	/
LH	446.96	414.2 ± 31.0	/	/
IH/LH	0.247	0.25	/	/
CFI	4.09	3.70 ± 0.94	4.24 ± 1.00	4.05 ± 0.66
MCFI	2.39	2.36 ± 0.41	2.42 ± 0.35	/
DCFI	1.71	/	1.75 ± 0.31	/
CI	0.58	/	0.61 ± 0.07	0.55 ± 0.05

**Figure 9:**
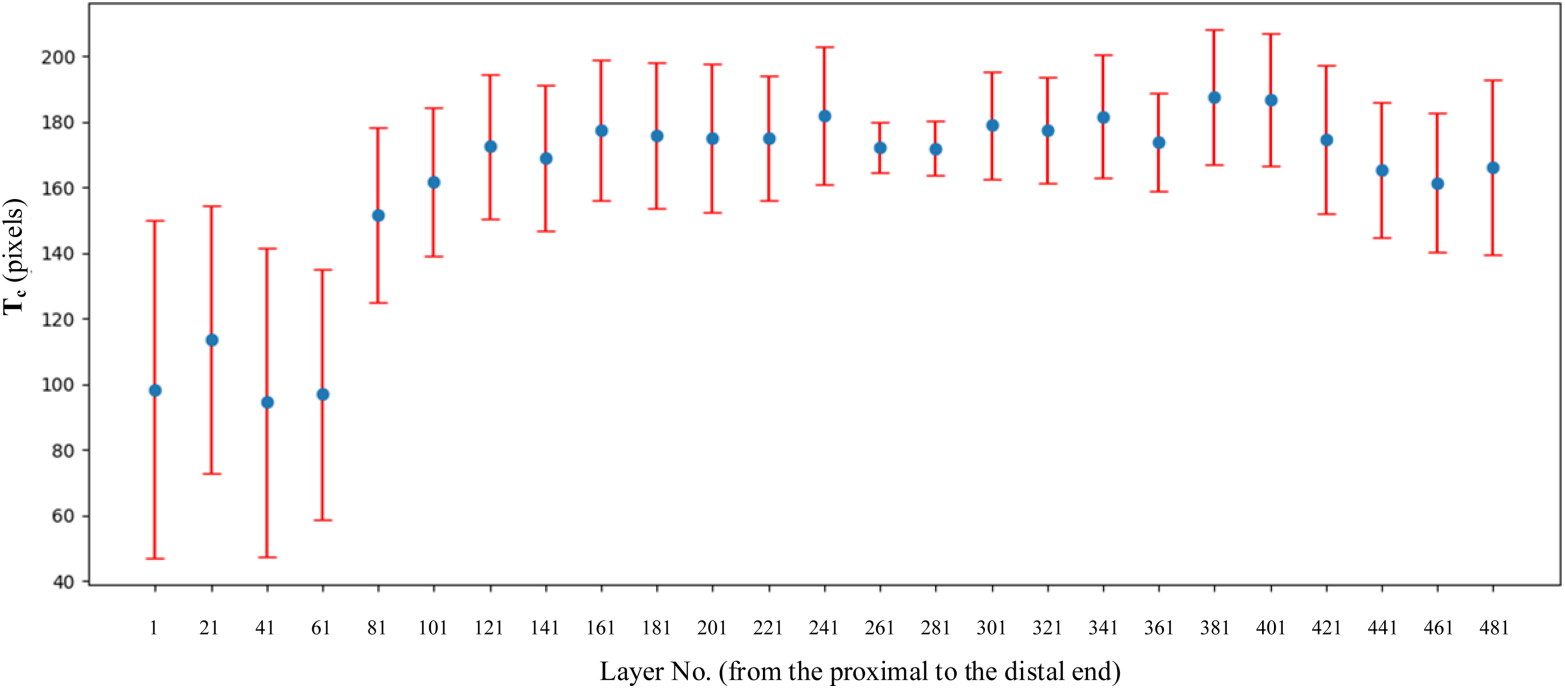
Variation of *T*_c_ on every layer. The selection of layer was 1 out of every 20 CT images.

From Figure 9, it can be observed that in the proximal femur, particularly in the metaphysis region, the cortical bone is significantly thinner than in the shaft region. This is consistent with the findings reported in the existing literature (40). This indicates that the shape and size of the prosthesis should be adapted to the anatomical structure of the femur, especially to match the morphology of the region with thin cortical bone, in order to prevent prosthesis loosening.

### 4.2 Morphological parameters defined in our sthdy

Combined with the parameters defined in Table 2, the values of basic intermediate variable parameters used in parameter calculation in Table 2 are shown in Table 6. The statistics for these parameters include the maximum value, minimum value, mean, and standard deviation.

**Table 6:** Morphological parameters of personalized femoral marrow cavity defined in this article.

	Total layers	Minimum value	Maximum value	Average	Standard deviation
*s*_c_	492	2,062	3,377	2,497.79	301.587
*s*_m_	492	412	4,658	1,233.51	1,038.959
*s*	492	2,868	7,996	3,731.30	1,239.042
*q*_1_	492	0.12	0.60	0.2921	0.13587
*q*_2_	492	0.1418	1.4749	0.478445	0.3490807

## 5 Discussion

### 5.1 Diameter parameters and prosthesis design

As can be seen from Table 5, the values of *T* + 20, *T* − 20 and *T*_0_ in this study were similar to those in references (23) and (25), and were less than those in the Noble (39) group and Rubin (41) group. This discrepancy suggests that when designing prostheses for patients, reference should not be made to prostheses imported from Western countries, but rather the design should be based on the medullary cavity data of the Chinese population.

Particularly noteworthy is that *T* − 20 and *T*_0_ were significantly smaller than the Noble group (39). The area 20 mm below the lesser trochanter represents the transition zone between the femoral shaft and the metaphysis. Histologically, this region exhibits a transition from spongy to compact bone. Morphologically, there is a marked narrowing at this junction. To allow the femoral shaft to pass through this narrow area and to achieve a press-fit in the proximal metaphyseal region, medullary reaming is usually necessary. However, over-reaming can compromise the load-bearing capacity of the proximally fixed femoral implant. For a medullary canal that is significantly narrowed to a funnel shape at *T* − 20, it is recommended that the femoral stem be designed with a narrower neck-shaft junction and a smaller anteroposterior diameter to ensure proper fit and stability.

Additionally, we are considering whether the differences between our *T* − 20 and *T*_0_ values and those of international groups are a result of using a fixed 40 mm interval as a marker for all subjects. Unfortunately, to date, there have been no reports confirming any negative effects of this fixed 40 mm selection. Therefore, in clinical practice, this method of fixed selection has consistently followed the approach used by Noble. Certainly, without sufficient sample data, it is possible that individual variations may account for such outcomes. In the future, we may investigate the correlation between medullary cavity parameters and the fixed selection of 20 mm above and below the lesser trochanter.

### 5.2 Ratio parameters and prosthesis design

Noble (39), a foreign scholar, proposed for the first time in 1988 to use CFI to describe the shape of the proximal medullary cavity of the femur. They measured and analyzed femur x-rays from 200 normal adults, obtaining a CFI value of 3.8 ± 0.74. According to the CFI value, the proximal femoral medullary cavity can be divided into chimney (CFI < 3.0), normal (3.0 ≤ CFI < 4.7), and champagne types (4.7 ≤ CFI < 6.5). Studies indicate that as the elderly age, CFI tends to decrease overall, resulting in a medullary cavity that becomes broader and straighter. Consequently, when designing femoral stem prostheses, preference should be given to larger, distally columnar stems or those with a distally wide rectangular shape. Designing a slender, tapered cylindrical stem at the distal end may make it challenging to achieve the desired three-point fixation, thereby compromising initial stability.

IH and T_mi_ determine the optimal length, distal diameter, and the depth at which the prosthesis should be implanted. CI is a parameter that measures the ratio of the thickness of the inner and lateral cortices of the femoral isthmus to the transverse diameter of the femoral isthmus. Its clinical significance lies in fracture risk assessment and bone density evaluation. A higher CI indicates that the femur has greater structural strength, which may reduce the risk of fractures. When using certain prosthesis stems that require distal medullary cavity expansion, it is important to design an appropriate prosthesis and consider the thickness of the distal cortex to avoid femoral shaft fractures due to excessive expansion. The Cortical Index can serve as a certain reference for the degree of medullary cavity expansion.

The femoral stem needs to match the metaphyseal end of the femoral shaft, while CFI describes the shape of the entire proximal femur, and MCFI describes the shape of the metaphyseal medullary cavity, which is crucial for the design of the femoral stem prosthesis. In this experiment, the value of MCFI is 2.39. Chen et al. (23) emphasizes that the value of MCFI has a significant correlation with age. When designing a proximally fixed prosthesis, we should fully consider the impact of age factors.

### 5.3 Morphological parameters defined in our study

**Figure 10:**
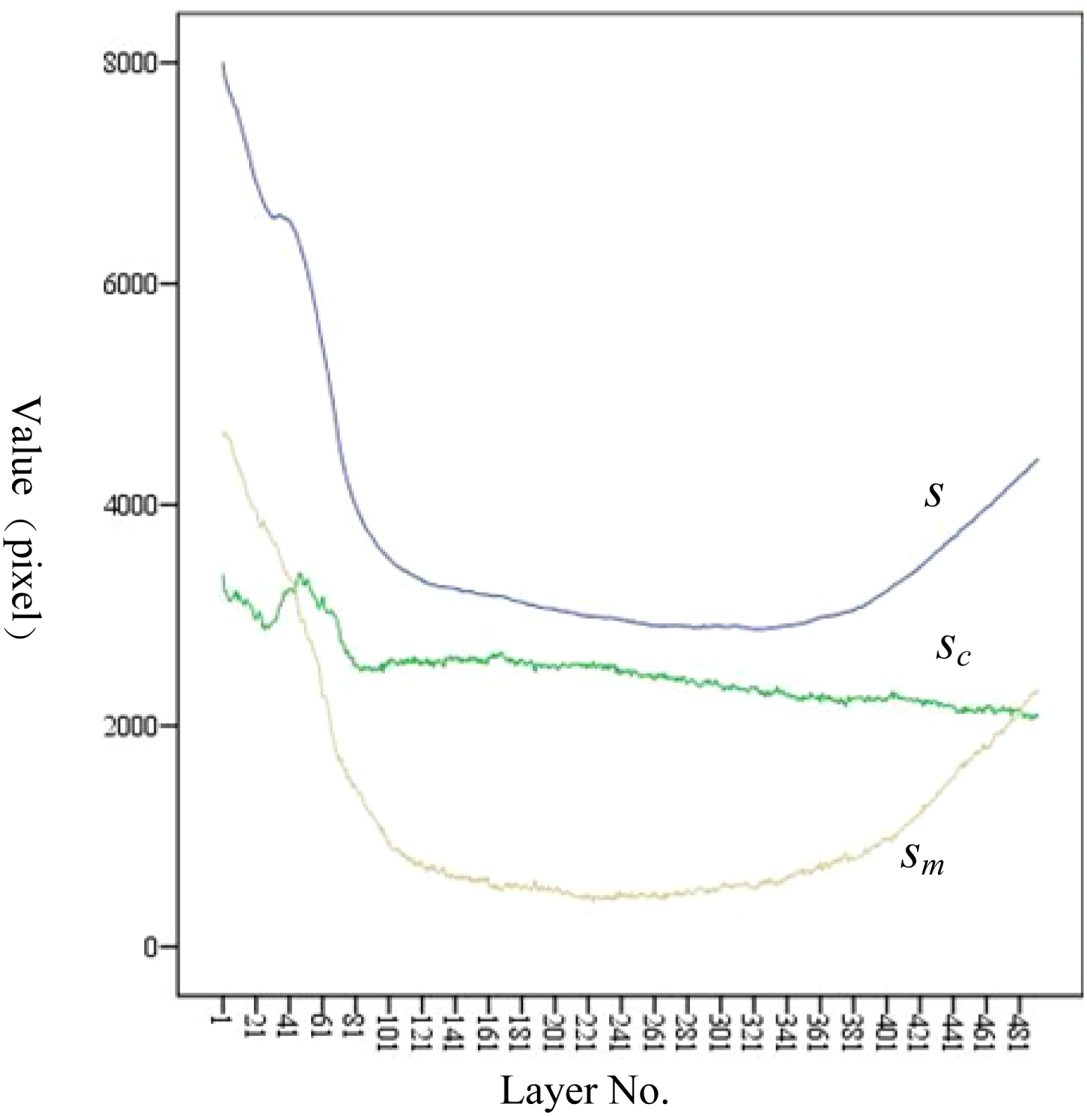
Variation trend of *s*, *s*_c_, and *s*_m_ at each layer. From the proximal end to the distal end, one of every 20 CT images is selected.

As shown in Figure 10, *s*_m_ shows a trend of first decreasing and then increasing. The position of the minimum value corresponds to the position of the isthmus. Combined with Tables 1, 5, the value of IH is 110.40 mm, and the value of LH is 446.96 mm. The ratio of IH to LH is calculated as 0.247, which is consistent with the ratio of 0.25 reported in the study by Chen et al. (23). Considering that the minimum often indicates the position of the isthmus, which plays an important role in guiding and limiting the placement of femoral prosthesis in clinical practice. Therefore, the isthmus is often used to mark the warning farthest position of the femoral stem in its implantation of the medullary cavity. To ensure good fixation, the length of the femoral stem should be selected with due consideration of the isthmus position. For the patients in this study, the stem of the femoral prosthesis should extend up to 24.7% of the total length of the femur for optimal fixation.

The analysis of *s*_c_ has the following clinical significance. First of all, *s*_c_ provides the clinician with an indication of the expanding medullary. To stabilize the femoral stem prosthesis, medullary cavity expansion should be conducted with careful consideration of sc at the isthmus to avoid puncturing the cortical bone. Secondly, *s*_c_ can be compared with the contralateral side during clinical diagnosis. If sc is significantly lower than that of the healthy femur, it indicates a decrease in bone strength.

**Figure 11:**
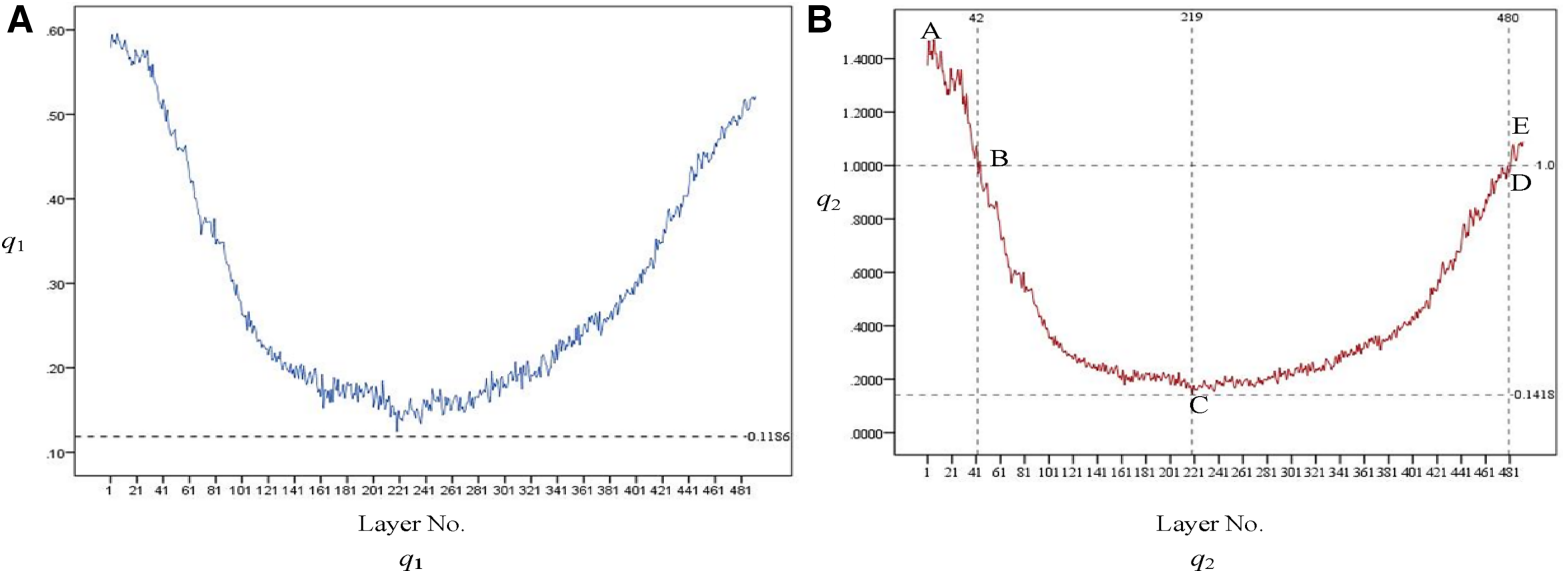
Variation trend of *q*_1_ [as shown in (**A**)] and *q*_2_ [as shown in (**B**)] at each layer. From the proximal end to the distal end, one of every 20 CT images is selected.

The variation trend of *q*_1_ at each layer is shown in Figure 11A. An increase in *q*_1_ indicates a tendency of medullary cavity enlargement, which significantly elevates the risk of fracture compared to that of normal healthy individuals. If there is an abundant data on healthy femoral bone marrow cavity, particular attention should be given to the average value of *q*_1_ at the marker cross section *C*_m0_, *C*_m0−20_, *C*_m0+20_, and *C*_m1_. This can provide more robust data support for clinical diagnosis.

The variation trend of *q*_1_ at each layer is shown in Figure 11B. *q*_2_ initially exhibits a downward trend and then an upward one after passing the lowest point, C. At points B and D, *q*_2_ equals 1, indicating that the area of the medullary cavity is equal to that of the cortical bone. *s*_m_ is larger than *s*_c_ on AB and DE and smaller than *s*_c_ on BD. This indicates that the proximal femoral cortex was thin in the bone and relatively thick in the isthmus. This suggests that, in the AB region, the thickness of the cortical bone is relatively small, and the medullary cavity is relatively large. Therefore, special attention should be given to the size of the femoral stem prosthesis to prevent it from penetrating the cortical bone. The area near point C is the narrowest part of the medullary cavity. Thus, it is essential to consider the farthest accessible position and size of the distal end of the femoral stem prosthesis.

*r*_1_ and *r*_2_ indicate the growth rate of internal medullary cavity of the lesser trochanter and the upper femoral shaft, respectively. In this experiment, *s*_m0+20_, *s*_m0−20,_ and *s*_m1_ was 3,313, 2,948, and 428, respectively. *r*_1_ can be calculated as *r*_1_ = (3,313 − 2,948)/2,948 = 0.124. *r*_2_ can be calculated as *r*_2_ = (3,313 − 428)/428 = 6.74. Therefore, during the design process of the femoral stem prosthesis, the narrowing of the femoral marrow cavity from proximal to distal should be fully considered.

### 5.4 Methods comparison

Measurement of femoral morphological parameters can be achieved through x-ray, CT scanning, and three-dimensional model reconstruction, each with its unique differences, advantages, and disadvantages. x-ray measurement is suitable for rapid screening and preliminary assessment, while CT scanning and three-dimensional model measurement are more appropriate for situations requiring precise measurement, such as preoperative planning. In our experiment, we employed a method that combines the use of Python v3.3 and Mimics v15.0 for calculating medullary cavity parameters. Additionally, we utilized SPSS v24.0 for data analysis. In our study, by introducing randomness into the problem of solving the ROIMC, the method simplifies and accelerates the calculation of the morphological parameters. The advantage of this approach is that it provides a probabilistic model for analyzing the femoral medullary cavity, allowing for a more comprehensive and nuanced understanding of its morphology.

In addition, we added the parameters listed in Table 2. These parameters provide more detailed and precise anatomical information than traditional Noble. This helps to more accurately assess the morphology of the femur, allowing for a more personalized fit in the implant design. In addition, the three-dimensional parameters can better reflect the anatomical differences between individuals. Overall, our proposed morphological parameters provide more detailed and personalized information than the traditional Noble classification system, leading to improvements in orthopaedic implant design, surgical planning, and patient care.

The proposed method was compared to existing methods, as shown in Table 7. It was found that the research method presented in this paper is characterized by high degree of automation, simplicity, flexibility, and efficiency, considering the multi-dimensional information.

**Table 7:** Comparison between method presented and traditional method.

	Lin KJ (22)	Chen HX (23)	Yan L (24)	Pi YG (25)	Soodmand E (26)	Noble (39)	Our work
Femoral data	CT	x-ray	CT	x-ray	3D model	x-ray	CT
Measuring tool	Software of CT machine	PACS	Software of CT machine	Onis v2.3	Geomagic studio v10.0	Customized computer routines	Python v3.3 and Mimics v15.0
Analysis tools	Microsoft Excel	SPSS v20.0	SPSS v19.0	SPSS v18.0	/	BMDP Statistical Software	SPSS v24.0
Degree of automation	Low	Medium	Low	Medium	Medium	High	High
Whether to measure in multiple dimensions	No	No	No	No	No	No	Yes

## 6 Conclusion

Accurate and full utilization of anatomical information contained in femoral CT data is critical to improving the quality of personalized femoral stem design. Considering the deficiencies related to obtaining medullary cavity morphological parameters, a Monte Carlo-based method was proposed to in-depth measure and analyze the parameters of the femoral bone marrow cavity. The main contributions of this study were as follows.

(1) By using Monte Carlo method, the problem of solving the ROIMC of the cross section of femoral marrow cavity without randomness was connected with a probability model to transform it into a problem of randomness. The results show that Monte Carlo-based method was simple and efficient to calculate the ROIMC of an irregular shape. It makes up for the lack of high-dimensional information acquisition in the existing medullary cavity morphological analysis
(2) The measurement results of defined parameters a supplement to the existing parameters, and therefore provide more reasonable scientific basis for comprehensively understanding the anatomical morphological changes of the femoral marrow cavity. This has important significance and application to clinical practice. This method also provides a reasonable and reliable method for the prediction of bone related diseases, such as osteoporosis.

The experimental results showed that the proposed method was simple, flexible, and efficient, and can provide innovative ideas, methods, and techniques for other type of bones. The limitation of this study is that it does not establish a mapping relationship between the morphological parameters of the femoral marrow cavity and the prosthesis parameters of the hip joint. This relationship will be explored further in future work.

## Data Availability

The original contributions presented in the study are included in the article/Supplementary Material, further inquiries can be directed to the corresponding author.
